# Efficacy of ultrasound-guided fascia iliaca compartment block after hip hemiarthroplasty: A prospective, randomized trial

**DOI:** 10.1097/MD.0000000000005018

**Published:** 2016-09-30

**Authors:** Seunguk Bang, Jihyun Chung, Jaejung Jeong, Hahyeon Bak, Dongju Kim

**Affiliations:** a Department of Anesthesiology and Pain Medicine; b Department of Orthopedic Surgery, College of Medicine, The Catholic University of Korea, Seoul; c Department of Surgery, College of Medicine, Chungbuk National University, Cheongju, Republic of Korea.

**Keywords:** analgesia, arthroplasty, fascia iliaca compartment block, fentanyl consumption, lumbar plexus, nerve block, ultrasonography

## Abstract

**Background:**

The fascia iliaca compartment block (FICB) provides an analgesic effect in patients with femur fractures. However, the postoperative pain after hip surgery is different from that after femur fracture, because of the difference in the degree and location of tissue trauma. Whether FICB provides effective postoperative analgesia in patients undergoing total hip arthroplasty is not well understood. Moreover, there is no prospective randomized study to evaluate FICB as a postoperative analgesia in hemiarthroplasty. Therefore, we performed a randomized and prospective study to determine the effect of FICB after hemiarthroplasty. The objective of this study was to compare the opioid consumption between patients who received intravenous patient-controlled analgesia (PCA) with and without FICB.

**Methods:**

Twenty-two patients aged 70 to 90 years who underwent bipolar hemiarthroplasty for femoral neck fracture were recruited and allocated randomly into 2 groups: FICB group (n = 11) and Non-FICB group (n = 11). All patients received spinal anesthesia with 10 mg of 0.5% hyperbaric bupivacaine. After surgery, the FICB was conducted using a modified technique with 0.2% ropivacaine (40 mL) under ultrasonographic guidance, and the intravenous PCA was administered to patients in both groups in the separate block room. The PCA was set up in the only bolus mode with no continuous infusion. The visual analog scale (VAS) and the opioid consumption were noted at 4, 8, 12, 24, and 48 hours postoperatively.

**Results:**

The VAS was similar in both groups. The fentanyl requirement at 4, 8, and 12 hours was low in the FICB group. The total amount of fentanyl required in the first 24 hours was 246.3 μg in the FICB group and 351.4 μg in the Non-FICB group. No patient developed any residual sensory-motor deficit during the postoperative period. Patients in the Non-FICB group had nausea (n = 2), and pruritus (n = 1), and 1 patient had nausea in the FICB group during postoperative 2 days.

**Conclusion:**

The FICB has a significant opioid-sparing effect in first 24 hours after hemiarthroplasty. This suggests that FICB is an effective way for multimodal analgesia in hip surgery.

## 1 Introduction

Fascia iliaca compartment block (FICB) is an anterior approach to the lumbar plexus. The pop technique using fascial click had a low success rate of 35% to 47%.^[1,2]^ However, as the FICB was performed under real-time ultrasound guidance, the success rate was increased to 82% to 87%, leading to an increased interest in FICB as a postoperative analgesia option for hip and knee surgical procedures.^[2,3]^

Some studies revealed that the FICB provides an analgesic effect in patients with femur fractures. Foss et al,^[4]^ in a double-blind randomized study, performed FICB in 48 patients with acute hip fractures. The FICB group was administered FICB with 1.0% mepivacaine and a placebo intramuscular injection of isotonic saline, whereas the morphine group was administered a placebo FICB with 0.9% saline and an intramuscular injection of 0.1 mg/kg morphine. Maximum pain relief was obtained in the FICB group. They concluded that the FICB reduced opioid consumption and was an effective and easily learned procedure. In addition, some studies reported that the FICB decreased the preoperative pain score and opioid consumption in patients with a femur fracture.^[5,6]^

Additionally, there are few studies where FICB provided better quality of position, reduced spinal anesthesia performance time, and less opioid analgesic consumption on the first postoperative day than intravenous opioid when spinal anesthesia was performed in patients with hip fracture.^[7,8]^

However, the postoperative pain after hip surgery including hemiarthroplasty and total arthroplasty was different from that of femur fracture, because the degree and location of tissue trauma were substantially different in patients with a femoral neck fracture and those with hip surgery. Moreover, established surgical treatments for displaced femoral neck fracture are mainly hemiarthroplasty and total hip arthroplasty (THA).^[9]^ Even though there are a few randomized studies on FICB in THA, whether FICB is an effective method for postoperative analgesia in THA is not well understood.^[10–12]^ Moreover, to the best of our knowledge, there is no prospective randomized study to evaluate the FICB as a postoperative analgesia in hemiarthroplasty. Therefore, we evaluated the effect of the FICB as a postoperative analgesia in hemiarthroplasty. Our hypothesis was that the FICB would decrease the opioid consumption during the first 24 hours after hemiarthroplasty.

## 2 Methods

We randomized patients in our randomized controlled trial comparing the efficacy of the FICB after hemiarthroplasty. This study was approved by the Catholic University Hospital Institutional Review Board, Daejeon, Korea (DC13EISI0075) and registered with the WHO Clinical Research Information Service (http://cris.nih.go.kr/cris, KCT0001450). Written informed consent was obtained from all patients enrolled in the study. Twenty-two patients with femoral neck fracture scheduled for bipolar hemiarthroplasty at our center in 2015 to 2016 were recruited.

The eligibility criteria included ages from 70 to 90 years, American Society of Anesthesiologists (ASA) physical status I to III, and undergoing bipolar hemiarthroplasty in our hospital. Exclusion criteria were clinically significant coagulopathy, infection at the injection site, allergy to local anesthetics, severe cardiopulmonary disease (≥ASA IV), body mass index > 35 kg m^−2^, diabetic or other neuropathies, patients receiving opioids for chronic analgesic therapy, contraindication to spinal anesthesia, and inability to comprehend VAS and patient-controlled analgesia (PCA) device.

Patients were randomly assigned to receive an FICB with 40 mL of ropivacaine 0.2% with epinephrine 5 μg mL^−1^ (FICB group) or not (Non-FICB group) by a computer-generated random number table method with randomized group information sealed in an opaque envelope, which were numbered and used sequentially. Randomization was achieved at the preanesthetic room by one of the research team not involved in the block procedure and evaluation (author JC).

Perioperative anesthesia management was according to our hospital routine protocol. An 18-gauge intravenous cannula was inserted in the hand in the general ward. After arrival in the operation room, we applied supplemental oxygen by facial mask at 5 L min^−1^ and standard monitoring (noninvasive blood pressure, electrocardiography, and pulse oximetry) throughout anesthesia. Patients were premedicated with intravenous midazolam (0.02 mg kg^−1^) and fentanyl (0.5 μg kg^−1^). In all patients, spinal anesthesia was performed in the sitting position. The midline and level of the L3–4 and L4–5 intervertebral spaces were identified, and spinal anesthesia was administered using 10 mg hyperbaric solution of 0.5% bupivacaine injected using 25-gauge Quincke needle (Spinocan, B. Braun Melsungen AG, Melsungen, Germany). Patients were immediately placed in the supine position. Spinal anesthesia was considered successful when a bilateral block to T12, as assessed by loss of cold (cold ice) and pain (a 23-gauge needle) sensations, was established 10 minutes after the intrathecal injection. If spinal anesthesia failed, such patients were administered general anesthesia and were excluded from this study. After additional 18-gauge intravenous cannulation on the contralateral ankle, the bipolar hemiarthroplasty was performed in all patients in nondependent lateral position by 1 hip surgeon with 20 years’ experience. During the surgery, sedation was induced using continuous propofol infusion. Propofol infusion was titrated to light sleep equivalent to 5 on the Ramsey scale, at which stage patients exhibit a sluggish response to a light glabellar tap or to a loud auditory stimulus during surgery.^[13]^ After surgery, we confirmed patient's mental status to be alert and checked whether they were able to communicate and obey commands. Then, the patient was transferred to a separate block room. After standard monitoring, patients in the FICB group underwent FICB using ultrasound (WS80A, Samsung Medicine, Seoul, Korea); then, intravenous PCA was started in all patients in both groups. Our standard protocol for postoperative multimodal analgesia was performed in both groups.

### 2.1 Block techniques

**Figure 1:**
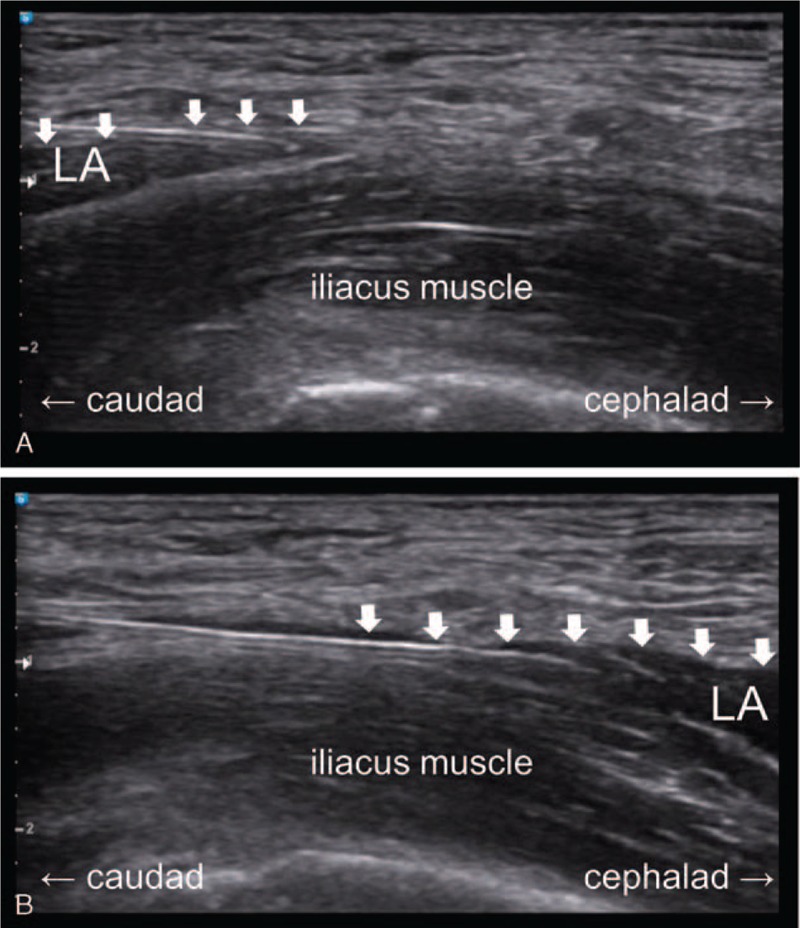
The heel-toe maneuver for needle visualization, when the needle is advanced. The needle is inserted through the skin using an in plane technique under ultrasonographic guidance. After confirming the passage of the needle through the fascia iliaca, a small amount normal saline is injected between the fascia iliaca and iliacus muscle. Longitudinal parasagittal view of the needle with its tip in position under fascia iliaca (A). Cephalad spread of the local anesthetic beneath the facia iliaca visualized in real time (B). For proximal spreading of local anesthetic, a small amount of solution is injected, followed by advancing the needle tip proximally using hydrodissection technique under ultrasonographic guidance, repeatedly. The fascia iliaca is indicated by line arrows. LA = local anesthetic.

In the supine position, the inguinal crease area was sterilized using betadine and chlorohexidine. After putting a 5 to 12 MHz linear probe parallel to the inguinal ligament on the inguinal crease, we found the femoral artery, fascia lata, fascia iliaca, iliacus muscle, and femoral nerve. From this view, after rotating the probe 90 to 135° counterclockwise, we made the probe parallel to the vertebrae axis. From this point, a 22G Tuohy needle (Taechang, Inc., Gongju, Korea), attached to an intravenous extension tubing between the needle and the syringe, was inserted along the plane and advanced toward the fascia iliaca and iliacus muscle. When the needle was advanced, the heel-toe maneuver was used for needle visualization. After confirming the passage of the needle through the fascia iliaca using fascial click and 2 mL of saline, we injected the prepared local anesthetics. If the needle was incorrectly placed, the needle was withdrawn to the region between the fascia iliaca and iliacus muscle. After injecting the normal saline, we used a hydrodissection technique under real-time ultrasound guidance (Fig. 1). In the hydrodissection technique, a small amount of local anesthetic was injected (1–2 mL); then, the needle was advanced to the proximal side for the proximal spreading of the local anesthetic. Through this process of hydrodissection, the needle is passed proximally, deep through the fascia iliaca and into the iliac fossa, moving only into the space created by fluid collection. The hydrodissection technique was repeated until the whole needle was inserted into the skin. The total volume was 40 mL of ropivacaine (AstraZeneca, Luton, UK) 0.2% with epinephrine 5 μg mL^−1^.

### 2.2 Nerve block assessment

We used 4 confirmatory steps for preventing the technical errors in the FICB: detecting a tactile, fascial “click” on needle passage through the fascia iliaca, observing immediate separation of the adjacent fascia iliaca and iliacus muscle or femoral nerve, observing the spreading of local anesthetics between the fascia iliaca and femoral nerve within 1 minute after injection of the local anesthetics, and more than 1 point at assessment of sensory block of the anterior thigh at postoperative 2 hours. Sensory blockade was graded according to a 4-point scale using ice: 0 = no block, 1 = hypoalgesia (decreased sensation of cold and pain compared to the opposite site), 2 = analgesia (patient can feel touch, not cold and pain), 3 = anesthesia (no sense of cold, pain, and touch). Sensory blockade of the femoral nerve was assessed on the anterior aspects of the thigh.^[14,15]^ When all of these criteria were met, the possibility of technical errors was excluded.

### 2.3 Multimodal analgesia protocol

All patients received intravenous ketorolac 30 mg at the end of surgery. Postoperative pain was assessed in the postanesthesia care unit (PACU) using a visual analog scale (VAS) in which a 0 indicated no pain and a 10 indicated the most severe pain. If the VAS was more than 4 points in the PACU, intravenous tramadol 25 mg was prescribed as a rescue analgesia.

Postoperative multimodal analgesia consisted of oral medication, intravenous analgesia, and rescue analgesics. Patients were prescribed oral celecoxib 200 mg twice daily for 7 days after surgery. In addition, intravenous fentanyl infusion was started in the PACU using a PCA pump (Hospira GemStar pump, Hospira Inc., Lake Forest, Illinois, USA). The PCA was programmed to deliver a bolus dose of 0.5 μg kg^−1^, without background infusion, with a lockout of 7 minutes, and a 4-hour limit of 4 μg kg^−1^. Nonetheless, if the pain was not controlled by the PCA throughout the postoperative period, intravenous tramadol 25 mg was prescribed as a rescue analgesia. When informed consent was obtained from patients before the day of surgery, we explained how to use intravenous PCA and how to evaluate the VAS. We also educated patients on VAS and the usage of PCA until they understood sufficiently, and instructed that the patient needs to push the bolus button if the pain score is 4 or more.^[16]^ The patients were instructed again in the preoperative waiting room.

The primary outcome was fentanyl consumption in the first 24 hours postoperatively; the secondary outcome was pain score using VAS. The pain score was investigated at postoperative 4, 8, 12, 24, and 48 hours in the 2 groups by an independent observer (author JJ), who was not involved in the block performance or patient assessment. The fentanyl consumption was investigated through the PCA program. The amount of tramadol injected as a rescue analgesia in the postoperative period was calculated as an equivalent dose of opioid (tramadol 25 mg = fentanyl 25 μg).^[17,18]^ During the injections, any vascular punctures, paresthesia, neural swellings, or other complications were recorded. The opioid-related side effects including nausea, pruritus, and respiratory depression were evaluated during postoperative 2 days. Neurologic complications such as allodynia, paresthesia, and numbness were also evaluated at postoperative 1 week.

### 2.4 Statistical analysis

In previous studies, the 24 hours morphine consumption was about 20 to 50 mg in hip surgery.^[10,12,14]^ The 24 hours fentanyl consumption was 300 to 500 μg because fentanyl is estimated to have about 100 times the potency of morphine as an opioid equivalent dose.^[17,18]^ In our pilot study, the 24 hours fentanyl consumption was found to be 387.62 ± 80.95 μg, which was similar to other studies. We decided to estimate a 25% (96.9 μg) reduction in fentanyl consumption to be clinically relevant. To show such a difference, a calculated sample size of 11 patients per group was required to provide us with a statistical power of 0.8 and a type 1 error of 0.05 for a 2-tailed test.

The normality of the data was evaluated using the Shapiro–Wilk test. For normally distributed data, the Student *t* test was used to compare the mean differences of variables between the groups. For variables with nonparametric distribution (age), the Mann–Whitney test was used to compare differences of the medians between the groups. Tests for significant differences between the groups were done with the *χ*^2^ test for categorical data (ASA, sex). A *P*-value of less than 0.05 was accepted as the level of significance. All data are presented as mean ± standard deviation (SD) and numbers. All statistical analyses were conducted using SPSS version 22.0 for Windows.

## 3 Results

**Figure 2:**
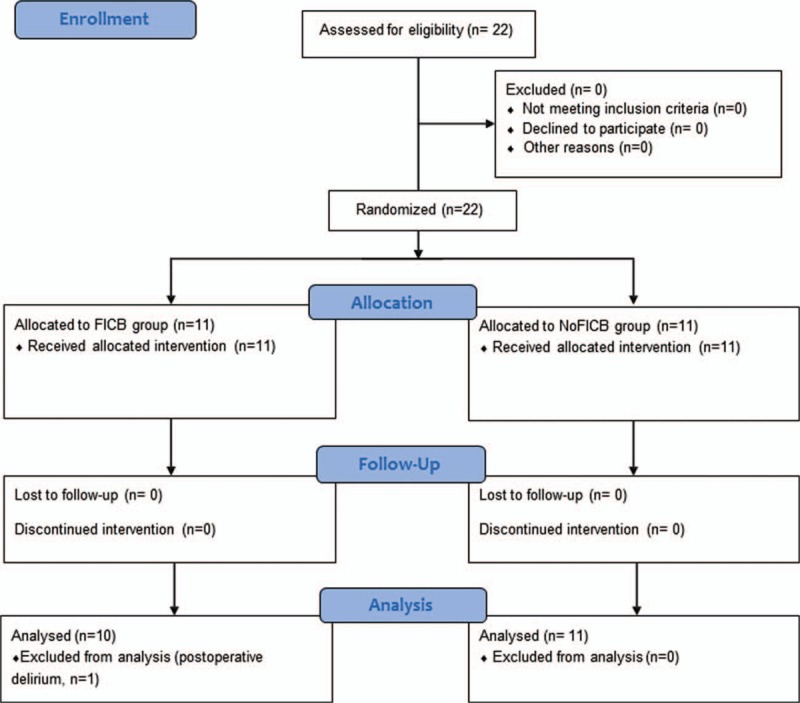
Consort diagram.

Twenty-two patients were enrolled in the study (Fig. 2). Data from 1 patient in the FICB group were excluded from the analysis due to postoperative delirium. Data from the remaining 21 patients were included in the final analysis. All patients in the 2 groups achieved sensory numbness at or above T12 dermatome after spinal anesthesia. All FICBs were completed with 40 mL of local anesthetic with an adequate spread of the local anesthetic as monitored by ultrasound. Moreover, there were no technical errors when we checked using the 4 confirmatory steps for preventing technical errors.

Patient characteristics and anesthetic-block-related data are presented in Table 1. There was no difference in pain scores between the 2 groups (Table 2). The amount of fentanyl required at 4 hours (18.5 mg vs 74.8 mg), 8 hours (36.4 vs 78.3 mg), and 12 hours (60.4 mg vs 80.5 mg) was low in the FICB group (*P* < 0.05). The total amount of fentanyl required in the first 24 hours was 246.3 ± 85.5 μg in the FICB group and 351.4 ± 87.53 μg in the Non-FICB group (*P* = 0.01) (Table 3).

**Table 1:**
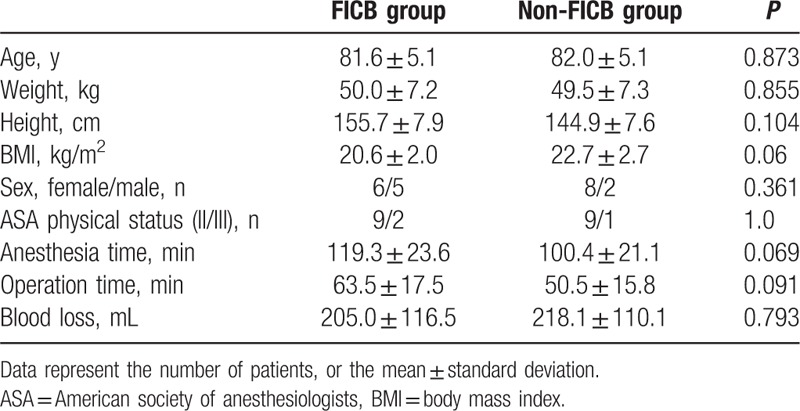
Demographic characteristics of patients in the 2 groups.

**Table 2:**
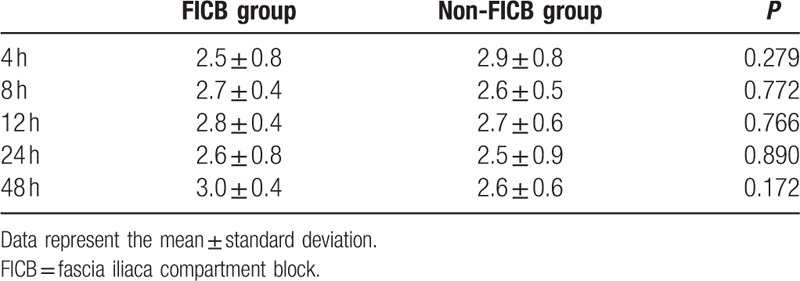
Visual analog scale.

**Table 3:**
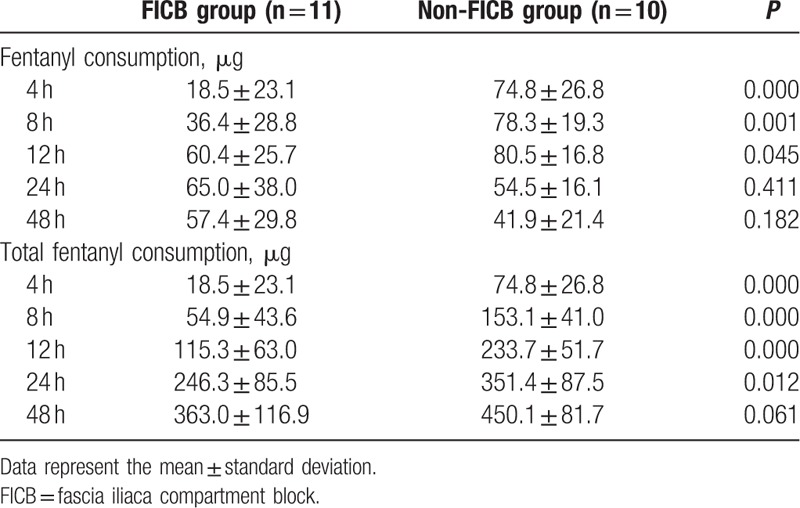
Amount of opioid consumption.

During the FICB, no paresthesia or vascular punctures were noted. No patient reported a VAS of more than 4 points or needed additional tramadol for analgesia in the PACU. None of the patients developed any residual sensory-motor deficit or complained of symptoms suggestive of neurologic injury at postoperative 2 days and 1 week. The patients in the Non-FICB group had nausea (n = 2), and pruritus (n = 1), and 1 patient had nausea in the FICB group during postoperative 2 days.

## 4 Discussions

The objective of this study was to evaluate the effect of the FICB as a postoperative analgesia in hemiarthroplasty. Our study shows that the FICB in hemiarthroplasty has less opioid consumption at 4, 8, and 12 hours in the postoperative period. In addition, the total fentanyl consumption during postoperative 24 hours was decreased in patients with the FICB.

The hip joint is innervated by multiple nerves. The anterior and anteromedial part in the capsule of the hip joint is innervated by the obturator nerve and femoral nerve. The posterior part is innervated by the sciatic nerve, which in addition to the articular branches from the nerves to the quadratus femoris muscle innervates the posteromedial part of the joint capsule. Moreover, the articular branches of the superior gluteal nerve innervate the posterolateral section of the joint capsule.^[19]^ Therefore, regional techniques to block the lumbar plexus such as the FICB, psoas compartment block, 3 in 1 block, and so on are the best choice for postoperative analgesia in hip surgery. Among them, the FICB is easy to perform and is accessed via a minimal risk approach to block the lumbar plexus. Previous studies show that the FICB has an analgesic effect after hip surgery, and their results are consistent with our conclusion. Goitia Arrola et al^[20]^ performed a prospective observational study of 41 patients undergoing total hip replacement surgery, and the FICB was effective in controlling early postoperative pain after surgery. Moreover, Krych et al^[21]^ reported that the FICB decreased the opioid consumption, and provided a high quality of pain relief and high overall patient satisfaction in patients with hip surgery.

However, Shariat et al^[12]^ reported no significant difference in postoperative pain score and 24 hours opioid consumption in 32 patients between FICB with 0.5% ropivacaine 30 mL and sham block with 0.9% normal saline 30 mL in THA.

In their study, a complete block of all 3 nerves including femoral nerve, obturator nerve, and lateral femoral cutaneous nerve were only 2 patients, and the incidence of a successful block of an individual nerve was 25% to 38%. This shows that the technique in their study is not sufficient to block the branches of the lumbar plexus such as femoral nerve, obturator nerve, and lateral femoral cutaneous nerve. This is why the proximal spreading of local anesthetics was not achieved because the infrainguinal technique and transverse plane were used rather than the longitudinal plane.^[22,23]^

When the FICB was performed, proximal spreading of the local anesthetic is an important factor because the aim of the FICB is to block the branches of the lumbar plexus. Therefore, our study used 2 ways including large volume and modified technique for ensuring the proximal spreading of local anesthetics.

First, we determined that the required volume of the local anesthetic was 40 mL. Helayel et al^[24]^ reported that the effective volumes of local anesthetics in the FICB capable of producing a block in 99% of cases were 36.6 mL (34.3–40.5) using ropivacaine. Moreover, several other studies used a large volume of 30 to 40 mL for proximal spreading in the FICB.

Second, the modified FICB technique with hydrodissection under real-time ultrasound guidance was used to maximize proximal spreading of local anesthetics.

In a cadaver study, the authors investigated the spread of a dye and nerve involvement in a cadaveric dye-injection model, using a modified ultrasound-guided technique. The lateral femoral cutaneous nerve, ilioinguinal nerve, and iliohypogastric nerve were more stained in the modified FICB than by the traditional method.^[10,25]^

Other clinical studies showed similar results to that of the cadaveric study. Dulaney-Cripe et al^[26]^ reported that 42 patients undergoing THA were subjected to the modified FICB under ultrasonographic guidance using 60 mL of 0.5% ropivacaine and infused continuously with 0.2% ropivacaine at the rate of 10 mL/h for postoperative analgesia. This modified FICB technique for the proximal spreading of local anesthetics decreased the postoperative pain score and hospital stay.^[26]^ Moreover, in another randomized study, the modified FICB using 40 mL of a local anesthetic resulted in the proximal spread of the local anesthetic and decreased the morphine consumption at 12 and 24 hours.^[10]^ Additionally, Kumar et al^[11]^ compared the analgesic efficacy of a suprainguinal approach (modified FICB) and an infrainguinal approach (traditional FICB) for postoperative analgesia. Forty patients scheduled for THA were recruited. The suprainguinal approach had a superior postoperative analgesic efficacy compared to the infrainguinal approach along with significantly less morphine consumption during first 24 hours.

This study has some limitations. First, we did not evaluate the innervated area of individual nerves. Patients were administered spinal anesthesia because most elderly people in Korea prefer regional anesthesia than general anesthesia due to fear of not recovering from general anesthesia. Therefore, we cannot decide the time of assessment and evaluation across the board. In addition, the evaluation of nerve block was not meaningful because the recovery time from spinal anesthesia was uncertain. Future studies on minimal effective volume for proximal spreading and relation between blocked nerve and analgesic effect are needed.

In conclusion, the FICB had significant opioid sparing effect in the first 24 hours in hemiarthroplasty. This suggests that modified FICB is an effective way for multimodal analgesia in hip surgery.

## Acknowledgment

Assistance with the study: none.

## References

[1] Capdevila X Biboulet P Bouregba M Comparison of the three-in-one and fascia iliaca compartment blocks in adults: clinical and radiographic analysis. *Anesth Analg* 1998; 86:1039–1044. 9585293 10.1097/00000539-199805000-00025

[2] Dolan J Williams A Murney E Ultrasound guided fascia iliaca block: a comparison with the loss of resistance technique. *Reg Anesth Pain Med* 2008; 33:526–531. 19258967 10.1016/j.rapm.2008.03.008

[3] Deniz S Atim A Kurklu M Comparison of the postoperative analgesic efficacy of an ultrasound-guided fascia iliaca compartment block versus 3 in 1 block in hip prosthesis surgery. *Agri* 2014; 26:151–157. 25551810 10.5505/agri.2014.76993

[4] Foss NB Kristensen BB Bundgaard M Fascia iliaca compartment blockade for acute pain control in hip fracture patients: a randomized, placebo-controlled trial. *Anesthesiology* 2007; 106:773–778. 17413915 10.1097/01.anes.0000264764.56544.d2

[5] Kumie FT Gebremedhn EG Tawuye HY Efficacy of fascia iliaca compartment nerve block as part of multimodal analgesia after surgery for femoral bone fracture. *World J Emerg Med* 2015; 6:142–146. 26056546 10.5847/wjem.j.1920-8642.2015.02.010 PMC4458475

[6] Wathen JE Gao D Merritt G A randomized controlled trial comparing a fascia iliaca compartment nerve block to a traditional systemic analgesic for femur fractures in a pediatric emergency department. *Ann Emerg Med* 2007; 50:162–171.171.e161. 17210208 10.1016/j.annemergmed.2006.09.006

[7] Yun MJ Kim YH Han MK Analgesia before a spinal block for femoral neck fracture: fascia iliaca compartment block. *Acta Anaesthesiol Scand* 2009; 53:1282–1287. 19650803 10.1111/j.1399-6576.2009.02052.x

[8] Diakomi M Papaioannou M Mela A Preoperative fascia iliaca compartment block for positioning patients with hip fractures for central nervous blockade: a randomized trial. *Reg Anesth Pain Med* 2014; 39:394–398. 25068412 10.1097/AAP.0000000000000133

[9] Bernstein J Ahn J In brief: fractures in brief: femoral neck fractures. *Clin Orthop Relat Res* 2010; 468:1713–1715. 20224957 10.1007/s11999-010-1295-7 PMC2865592

[10] Stevens M Harrison G McGrail M A modified fascia iliaca compartment block has significant morphine-sparing effect after total hip arthroplasty. *Anaesth Intensive Care* 2007; 35:949–952. 18084988 10.1177/0310057X0703500615

[11] Kumar K Pandey RK Bhalla AP Comparison of conventional infrainguinal versus modified proximal suprainguinal approach of Fascia Iliaca Compartment Block for postoperative analgesia in Total Hip Arthroplasty. A prospective randomized study. *Acta Anaesthesiol Belg* 2015; 66:95–100. 26767235

[12] Shariat AN Hadzic A Xu D Fascia lliaca block for analgesia after hip arthroplasty: a randomized double-blind, placebo-controlled trial. *Reg Anesth Pain Med* 2013; 38:201–205. 23558369 10.1097/AAP.0b013e31828a3c7c

[13] Ramsay MA Savege TM Simpson BR Controlled sedation with alphaxalone-alphadolone. *Br Med J* 1974; 2:656–659. 4835444 10.1136/bmj.2.5920.656 PMC1613102

[14] Techasuk W Bernucci F Cupido T Minimum effective volume of combined lidocaine-bupivacaine for analgesic subparaneural popliteal sciatic nerve block. *Reg Anesth Pain Med* 2014; 39:108–111. 24496169 10.1097/AAP.0000000000000051

[15] Tran DQ Dugani S Pham K A randomized comparison between subepineural and conventional ultrasound-guided popliteal sciatic nerve block. *Reg Anesth Pain Med* 2011; 36:548–552. 22005661 10.1097/AAP.0b013e318235f566

[16] DeLoach LJ Higgins MS Caplan AB The visual analog scale in the immediate postoperative period: intrasubject variability and correlation with a numeric scale. *Anesth Analg* 1998; 86:102–106. 9428860 10.1097/00000539-199801000-00020

[17] Lehmann KA [Tramadol in acute pain]. *Drugs* 1997; 53 suppl 2:25–33. 9190322 10.2165/00003495-199700532-00007

[18] Pereira J Lawlor P Vigano A Equianalgesic dose ratios for opioids. A critical review and proposals for long-term dosing. *J Pain Symptom Manage* 2001; 22:672–687. 11495714 10.1016/s0885-3924(01)00294-9

[19] Birnbaum K Prescher A Hessler S The sensory innervation of the hip joint—an anatomical study. *Surg Radiol Anat* 1997; 19:371–375. 9479711 10.1007/BF01628504

[20] Goitia Arrola L Telletxea S Martinez Bourio R [Fascia iliaca compartment block for analgesia following total hip replacement surgery]. *Rev Esp Anestesiol Reanim* 2009; 56:343–348. 19725341 10.1016/s0034-9356(09)70406-2

[21] Krych AJ Baran S Kuzma SA Utility of multimodal analgesia with fascia iliaca blockade for acute pain management following hip arthroscopy. *Knee Surg Sports Traumatol Arthrosc* 2014; 22:843–847. 24061718 10.1007/s00167-013-2665-y

[22] Vaughan B Manley M Stewart D Distal injection site may explain lack of analgesia from fascia iliaca block for total hip. *Reg Anesth Pain Med* 2013; 38:556–557. 24153047 10.1097/AAP.0000000000000011

[23] Murgatroyd H Forero M Chin KJ The efficacy of ultrasound-guided fascia iliaca block in hip surgery: a question of technique? *Reg Anesth Pain Med* 2013; 38:459–460. 10.1097/AAP.0b013e31829d27fa 23970049

[24] Helayel PE Lobo G Vergara R [Effective volume of local anesthetics for fascia iliac compartment block: a double-blind, comparative study between 0.5% ropivacaine and 0.5% bupivacaine]. *Rev Bras Anestesiol* 2006; 56:454–460. 19468591 10.1590/s0034-70942006000500003

[25] Hebbard P Ivanusic J Sha S Ultrasound-guided supra-inguinal fascia iliaca block: a cadaveric evaluation of a novel approach. *Anaesthesia* 2011; 66:300–305. 21401544 10.1111/j.1365-2044.2011.06628.x

[26] Dulaney-Cripe E Hadaway S Bauman R A continuous infusion fascia iliaca compartment block in hip fracture patients: a pilot study. *J Clin Med Res* 2012; 4:45–48. 22383926 10.4021/jocmr724w PMC3279500

